# How I do it: feasibility of a new ultrasound probe fixator to facilitate high quality stress echocardiography

**DOI:** 10.1186/s12947-018-0124-0

**Published:** 2018-03-27

**Authors:** O. A. E. Salden, W. M. van Everdingen, R. Spee, P. A. Doevendans, M. J. Cramer

**Affiliations:** 1 Department of Cardiology, University Medical Centre Utrecht, P.O. Box 85500, 3508 GA Utrecht, The Netherlands; 2 Department of Cardiology, Maxima Medisch Centrum, Veldhoven, The Netherlands; 3 Netherlands Heart Institute, Central Military Hospital, Utrecht, The Netherlands

**Keywords:** Stress echocardiography, Ultrasound, Probe, Fixation

## Abstract

**Background:**

Stress echocardiography (SE) has recently regained momentum as an important diagnostic tool for the assessment of both ischemic and non-ischemic heart disease. Performing SE during physical exercise is challenging due to a suboptimal patient position and vigorous movements of the patient’s chest. This hampers a stable ultrasound position and reduces the diagnostic performance of SE. A stable ultrasound probe position would facilitate producing high quality images during continuous measurements. With Probefix (Usono, Eindhoven, The Netherlands), a newly developed tool to fixate the ultrasound probe to the patient’s chest, stabilization of the probe during physical exercise is possible.

**Implementation and results:**

The technique of SE with the Probefix and its’ feasibility are evaluated in a small pilot study. Probefix fixates the ultrasound probe to the patient’s chest, using two chest straps and a fixation device. The ultrasound probe position and angle may be altered with a relative high degree of freedom. We tested the Probefix for continuous echocardiographic imaging in 12 study subjects during supine and upright ergometer stress tests. One patient was unable to perform exercise and in two study subjects good quality images were not achieved. In the other patients (82%) a stable probe position was obtained, with subsequent good quality echocardiographic images during SE.

**Conclusion:**

We have demonstrated the feasibility of the Probefix support during ergometer tests in supine and upright positions and conclude that this external fixator may facilitate continuous monitoring of cardiac function in a group of patients.

**Electronic supplementary material:**

The online version of this article (10.1186/s12947-018-0124-0) contains supplementary material, which is available to authorized users.

## Background

Stress echocardiography (SE) is a non-invasive and cost-effective tool to test myocardial function and hemodynamics during exercise [1]. It has most frequently been applied for the assessment of known or suspected ischemic heart disease, and has recently regained momentum as an important diagnostic tool [2]. The indications for the use of SE in non-ischemic heart disease are continuously evolving, as a consequence SE has also become widely implemented to assess various conditions other than ischemic heart disease, such as the assessment of systolic or diastolic heart failure, valvular heart disease, congenital heart disease, athletes’ hearts, and the presence of inotropic contractile reverse in patients eligible for cardiac resynchronization therapy (CRT) [1, 3, 4].

SE during physical exercise is closest to normal physiological conditions as it preserves the integrity of the electromechanical response and provides valuable information about the patients’ functional status. Pharmacological stress testing, contrastingly, does not replicate the complex hemodynamics and neurohormonal changes that are triggered by exercise [3, 5–7]. SE during physical exercise requires a treadmill or ergometer with dedicated equipment for exercise protocols. Unfortunately, physical exercise hampers a stable ultrasound probe position and echocardiographic image quality [8]. During physical exercise, the thorax moves due to vigorous cycling or running and heavy breathing. Both movements hamper a stable ultrasound probe position with manual application of the ultrasound probe. Additionally, due to the patient’s position during exercise induced SE, obtaining high quality images is challenging. Yet, image quality is imperative for obtaining an accurate diagnosis, as anomalies can be subtle. Consequently, SE is often performed directly after peak exercise, to reduce the motion artefacts caused by exercise. However, according to previous trials that assessed the diagnostic accuracy of exercise testing, continuous SE during exercise is superior to post-exercise testing as it can detect even small, quickly reversible wall motion abnormalities. Post-exercise testing, on the other hand, can miss important information about the existence, extension, and location of ischemia [5, 6].

SE during physical exercise would be more feasible and easy if a stable ultrasound probe position can be maintained throughout the test, whilst at the same time high quality images are being produced. With Probefix (Usono, Eindhoven, The Netherlands), a newly developed tool to fixate the ultrasound probe to the patient’s chest, the probe position is stabilized during echocardiography. Thereby Probefix enables stable and continuous echocardiographic measurements over time. Probefix additionally removes the necessity for sonographers to manually hold the probe during continuous stress testing, thereby enabling hands-free continuous measurements. We therefore believe that this device has the potential to improve the implementation of SE. In the present paper, we report the technique of SE with the Probefix and test its’ feasibility.

## Implementation

### Probefix

**Fig. 1:**
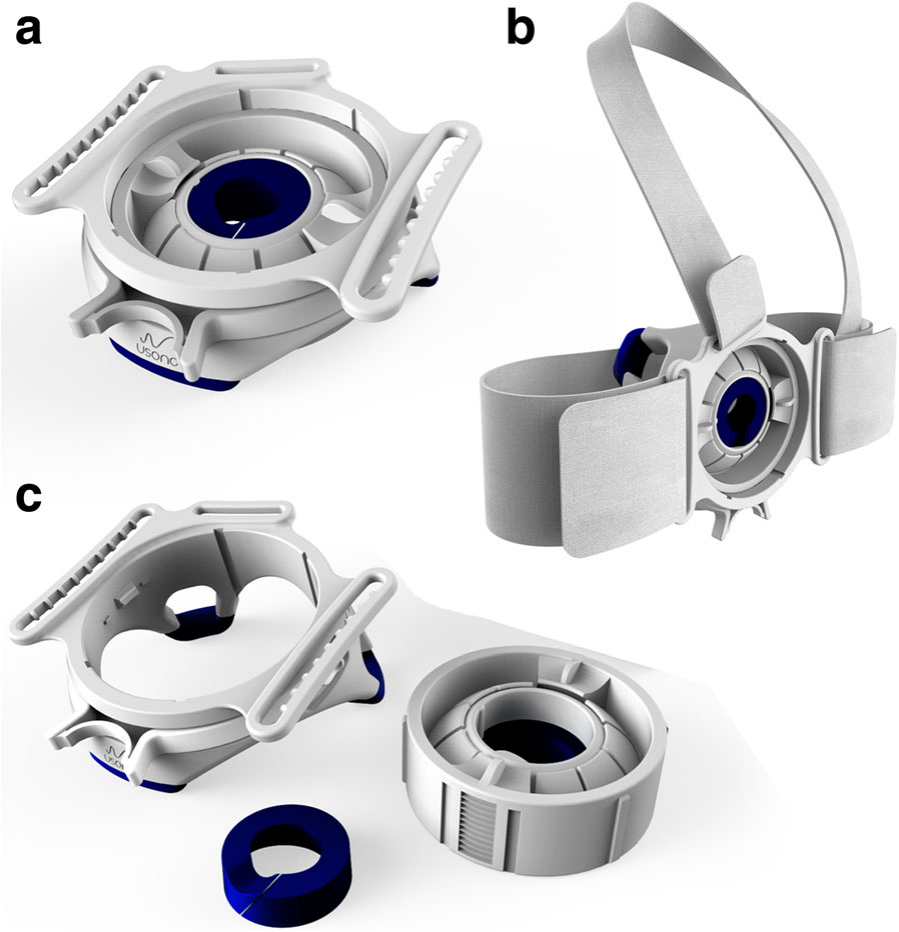
Overview of the Probefix. Overview of the Probefix and its specific parts. Panel (**a**) shows the mounted Probefix. The device can be attached to the patient’s chest with two chest straps (Panel **b**). Panel (**c**) shows a demounted version of the device. The external ring has three loops to attach the flexible chest straps, The internal holder fixates the ultrasound probe with the blue elastic ring, while the probe angle can be altered with a relative high freedom of degrees in any direction

**Fig. 2:**
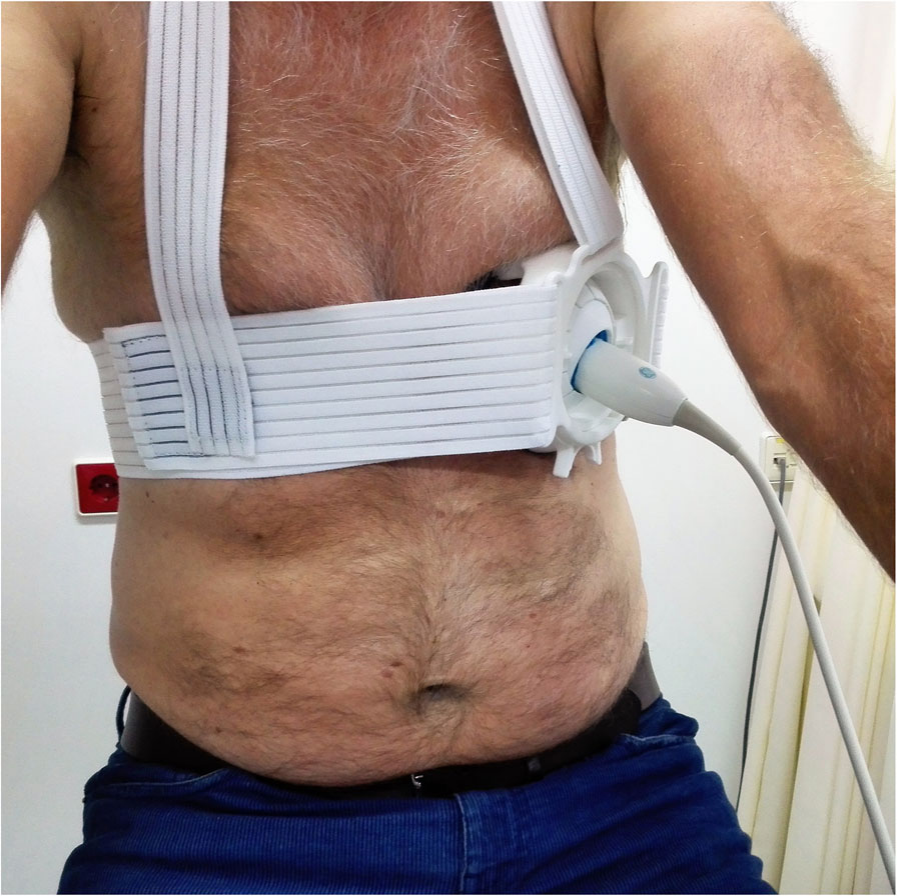
Apical fixation of the Probefix for echocardiography. The ultrasound probe (M5Sc, GE Healthcare, Milwaukee, USA) is fixated to the patient’s chest with the Probefix. The patient is sitting upright on the bicycle ergometer. First the optimal apical four chamber position is searched for without the fixator. Next, the probe is placed in the Probefix, which is then fixated to the patient by attaching the chest straps

Probefix is a non-invasive tool which provides lengthy and stable fixation of an ultrasound probe to the body. The Probefix consists of several parts that together enable fixation of the ultrasound probe to the patient’s chest for continuous echocardiographic examination (Fig. 1). The device is attached to the patient’s chest with two chest straps (Fig. 2). The horizontal and vertical chest strap may be adjusted to the patients’ chest size by the velcro tape. The external holder of the fixator has three rings to attach the flexible chest straps. The external holder has three silicon feet by which it is placed onto the patient’s chest. Within the external holder an internal holder fixates the ultrasound probe. Nonetheless, the probe angle can be altered with a relative high freedom of degrees in any direction, thus enabling rotating from, for example, the apical four chamber to two- and three chamber position. The internal holder can also be positioned up- and downward in the external ring, thereby affirming the probe towards the chest. The probe itself is held in the internal ring by a blue elastic ring, which is moulded to match the shape of the specific ultrasound probe. By using different elastic rings, ultrasound probes of different sizes and manufacturers can be fixated in the universal Probefix.

### Set up

**Fig. 3:**
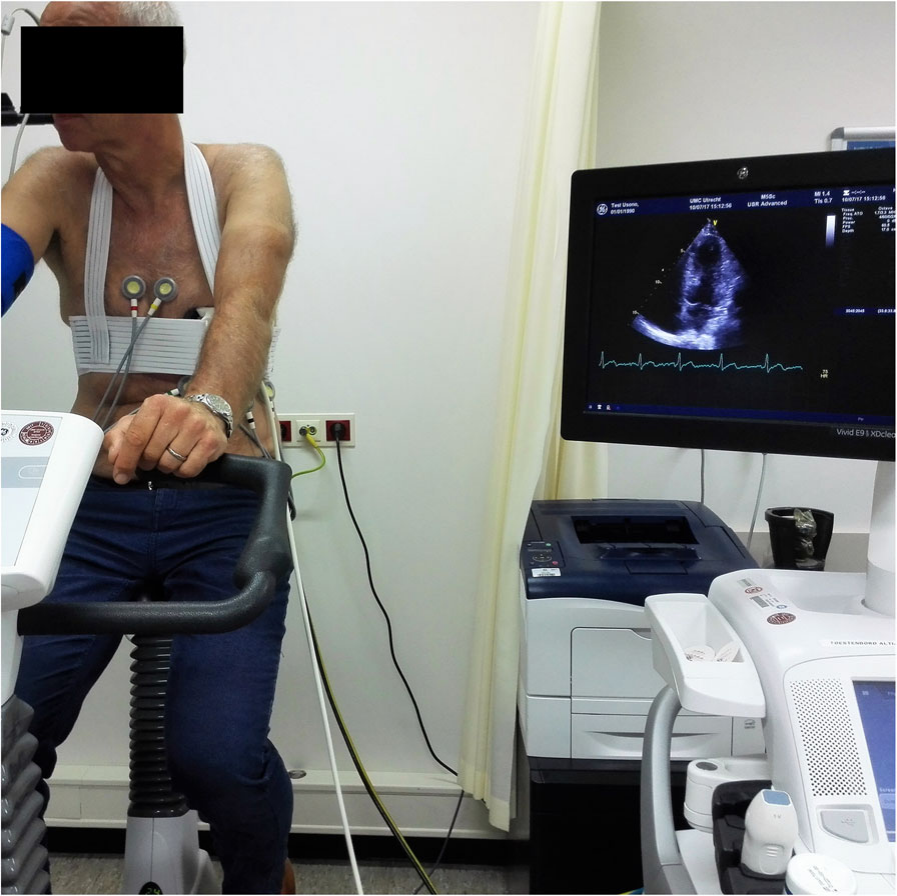
Upright bicycle ergometer test with stress echocardiography. Stress echocardiography during upright bicycle ergometer test with Probefix. The CASE ergometer machine (GE Healthcare, Milwaukee, US) is connected to the Ergoline bicycle (Ergoline GmbH, Bitz, German). The GE Vivid9 ultrasound machine is positioned on the left side of the patient to facilitate apical positioning of the ultrasound probe (M5Sc, GE Healthcare, Milwaukee, USA). With the ultrasound probe fixated in the apical position, continuous monitoring of the apical four chamber view is displayed on the echocardiographic monitor (Vivid9, GE Healthcare, Milwaukee, USA). Simultaneous electrocardiographic (ECG) monitoring is performed during an exercise protocol. See the online movie for an overview and results of the upright bicycle test with echocardiography using the Probefix (Additional file 1)

**Fig. 4:**
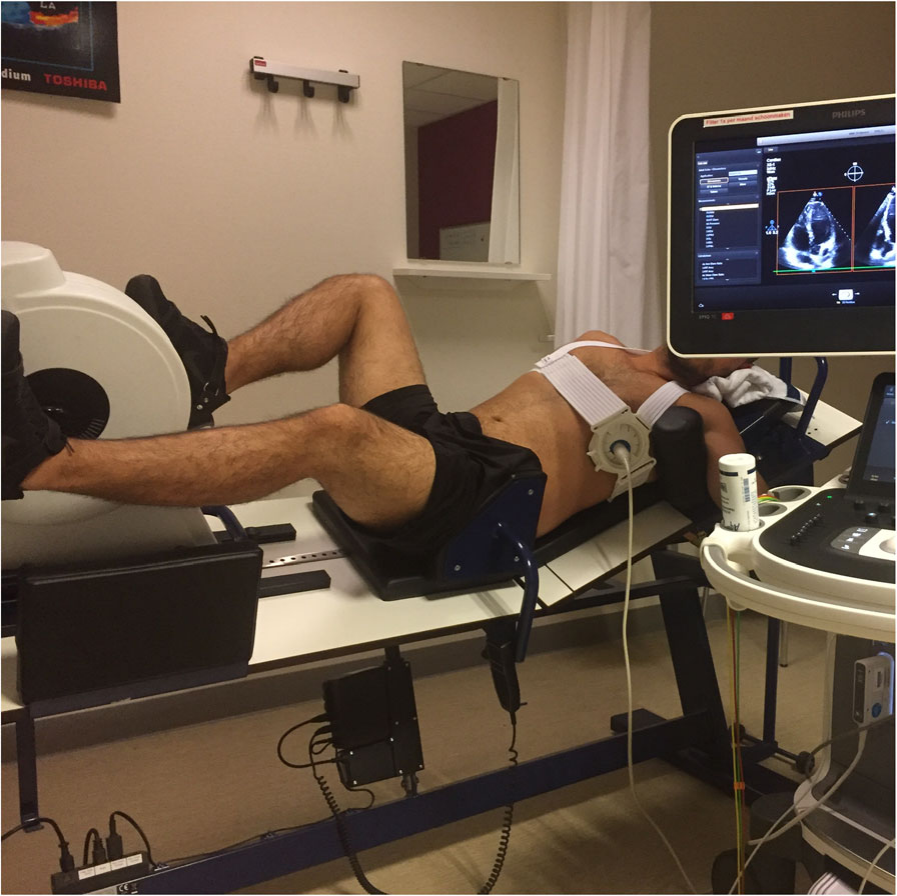
Supine bicycle ergometer test with stress echocardiography. Example of stress echocardiography with a patient lying on a supine bicycle ergometer. The table is tilted towards the left, to optimize the image quality of the apical four chamber view. The probe (X5-1, Philips Medical Systems, Best, The Netherlands) is fixated with Probefix and angulated in the optimal position. See the online movie for an overview of the supine bicycle test with echocardiography using the Probefix (Additional file 2)

Stress echocardiography requires a treadmill or bicycle ergometer with a dedicated system for stress protocols. The ergometer can be either upright or supine, depending on the available equipment (Figs. 3 and 4). During bicycle stress tests, a 12-lead ECG can be recorded simultaneously, combining a standard ergometer test and stress echocardiography (Fig. 3). Alongside the exercise module, an echocardiographic machine is positioned. Connected to the ultrasound machine is a conventional 2D or 3D brightness mode (B-mode) ultrasound probe.

Before positioning the ultrasound probe at the ideal acoustic window, the probe is loosely connected to the Probefix, without fixating the device to the patient’s body. This set up, allows for accurate, manual positioning of the ultrasound probe at the optimal position. When the desired acoustic window is identified, the Probefix can be fixated to the patient’s chest with the two Velcro straps. The horizontal chest strap is positioned first and the vertical chest strap is fixated next. Both should be firmly attached to the patient’s chest. The internal holder of the Probefix can then be pushed towards the chest to achieve a firm position, while rotating the ultrasound probe is still feasible.

### Measurements

During stress echocardiography, 2D or 3D B-mode images of the left ventricle may be obtained during various stages of physical exercise. Alternatively, pulsed wave or continuous wave Doppler recordings may be obtained over the cardiac valves. The ultrasound probe can be rotated and angulated to acquire all desired imaging planes (e.g. the apical two chamber, apical three chamber or apical four chamber view). Several parameters can be assessed, such as, ventricular function, valvular and subvalvular gradients and regurgitant flows. Implementation of a 3D ultrasound probe is even more convenient, as multiple 2D imaging planes can be recorded without rotating the probe.

### Pilot study set up

We performed SE with the Probefix in healthy volunteers and in patients from our outpatient clinic. Exclusion criteria were pharmacological stress testing and the necessity to perform imaging in both apical and parasternal view, because during stress test it is very challenging to relocate the Probefix. All patients that received exercise induced SE, for the assessment of valvular disease or CRT device optimization in January and February 2018 were asked for participation (*n* = 6). In addition we asked six healthy volunteers (colleagues) to undergo SE with Probefix. All volunteers signed informed consent prior to stress testing. Stress tests were performed at supine or upright bicycle ergometer and stress protocol were adjusted to the subjects physical fitness (Figs. 3 and 4). Study subject were tested until maximal exercise levels were achieved. In the healthy volunteers we performed imaging in either short axis view or apical view (depending on the best image quality) and we assessed whether during SE the image quality was sufficient for wall motion analysis. In patients from our outpatient clinic we used local imaging protocols according to the objectives of the diagnostic test.

Stress tests were performed on an Ergoline bicycle (Ergoline GmbH, Bitz, German) ergometer. For echocardiographic examination GE Vivid7 or Philips iE33 ultrasound machines were used, with 2D echo probes (M5Sc, GE Healthcare, Milwaukee, USA and X5-1, Philips Medical Systems, Best, The Netherlands). The study complied with the Declaration of Helsinki and the protocol was issued by the local Medical Research Ethics Committees (MREC/METC) as non-WMO (Medical Research Involving Human Subjects Act) research.

## Results

**Fig. 5:**
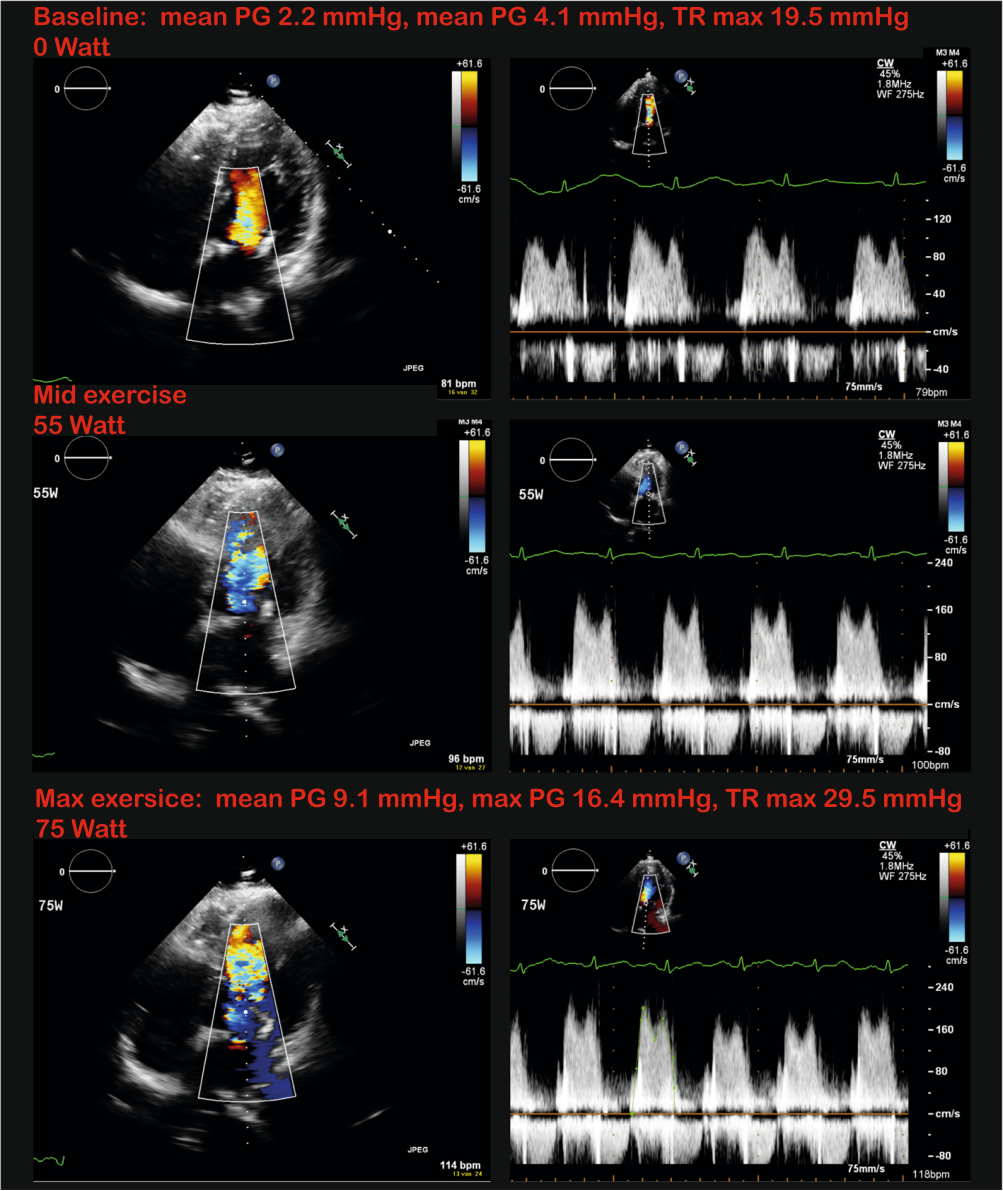
Stress echocardiography of mitral valve. SE performed with the Probefix in patient six. The patient had a history of mitral valve plasty due to P2 leaflet prolapse. Patient experienced progressive dyspnoea, fatigue and weight gain after surgery. SE was performed to assess the mitral valve pressure gradient during exercise, which was not elevated. At baseline and at peak exercise, right ventricular filling pressures were also assessed with continuous wave Doppler of the tricuspid valve

We performed SE in six heathy subjects and six patients of which a detailed description is given in Table 1. One patient was not able to perform exercise and was therefore excluded from further analysis. Subjects that were included in the feasibility study were 55% male, had a mean age of 42 ± 21 year, and a mean body mass index of 25 ± 5. Most outpatient subjects were tested for the evaluation of valvular disease (Fig. 5), while in one patient SE was performed for echocardiography-guided CRT device optimization. In two study subject, the image quality at baseline was insufficient to test Probefix. In all other subjects, the acoustic windows remained of good quality during increased load, and the echo probe remained stable at the fixated position. There was no repositioning necessary. The application of Probefix therefore was feasible in 9 out of 11 subjects (82%).

**Table 1:** Patients overview

Subject	Age	Gender	BMI	Indication	Stress test	Echo view	Quality baseline	Quality mid exercise	Quality max exercise	Probefix feasible
Patient 1	76	male	29.3	gradient MV	supine	AP4CH	good	good	good	yes
Patient 2	80	male	26.7	gradient MV	supine	AP4CH	good	good	n/a	n/a
Patient 3	34	female	21.3	device optimization in CRT patient	Supine	AP4CH	moderate	moderate	moderate	yes
Patient 4	82	female	33.2	gradient AoV	Supine	AP5CH	good	good	good	yes
Patient 5	19	female	22.2	gradient AoV	Supine	AP5CH, AP3CH	poor	n/a	n/a	no
Patient 6	43	female	35.2	gradient MV	Supine	AP4CH	good	good	good	yes
Volunteer 1	58	male		quality check	Upright	AP4CH	good	good	n/a	yes
Volunteer 2	35	male	23.1	quality check	Supine	AP4CH	good	good	good	yes
Volunteer 3	28	male	20.5	quality check	Supine	PSAX/AP4CH	poor	n/a	n/a	no
Volunteer 4	27	male	23.5	quality check	Supine	AP4Ch	moderate	moderate	moderate	yes
Volunteer 5	28	male	24.9	quality check	Supine	AP4CH	good	good	good	yes
Volunteer 6	27	female	21.1	quality check	Supine	AP4CH	good	good	good	yes

An overview of patients and volunteers that participated in the pilot study. Age, gender, body mass index (*BMI*), indication for stress test and feasibility of the Probefix is displayed. *MV* mitral valve, *AoV* aortic valve, *AP3CH* apical three chamber, *AP4CH* apical four chamber, *AP5CH* apical five chamber, *PSAX* parasternal short axis

Fixating Probefix to the patient at the best echocardiographic window took less than 5 min in all patients. Both supine and upright testing resulted in good quality echocardiographic images during exercise as is visualized in the two additional movie files (see Additional files 1 and 2).

## Discussion

We have demonstrated the feasibility of the Probefix in the field of SE for performing continuous, hands-free echocardiographic monitoring over time. In 82% of subjects, we acquired good quality acoustic windows, which remained of sufficient quality during increased load, without the need for repositioning the probe. Attaching the device to the patients was rapidly performed.

### Strengths and limitations

The inherent limitations associated with a small, pilot study should be acknowledged. Nevertheless, this study is the first to describe a new technique for facilitating continuous ultrasound measurements. The superiority of continuous echocardiographic measurements during exercise over post-exercise testing has previously been demonstrated by the studies of Dagianti et al. and Badruddin et al. [5, 6].

We demonstrate the feasibility of Probefix in five out of six healthy volunteers and four out of five patients from our outpatient clinic. There are however also limitations to its’ implementation. Firstly, due to the fixation of the echo probe, it is challenging to change from e.g. the parasternal view to the apical view during exercise testing. Fixating two Probefix devices to the patients’ chest could be a solution to this problem, however this is not possible in all patients due to lack of space on the patients’ thorax. Secondly, due to poor baseline echocardiographic windows, SE with Probefix will not be achievable in all patients. As a result of these limitations we believe that the primary application of the Probefix are echocardiographic imaging protocols in which only one view (either apical or parasternal) is required. Consequently, patients with valvular disease and patients whom undergo diastolic function testing are especially suitable for SE with the Probefix. In this patient group we strongly believe that Probefix facilitates continuous monitoring of cardiac function during SE. Because Probefix removes the necessity for sonographers to manually hold the probe during continuous stress testing, this could also reduce the risk of repetitive strain injuries. However more research is needed to test this hypothesis. As Probefix is a new device scientific proof of its added value is of importance. This article explores the possibilities of Probefix in the field of (stress) echocardiography. A trial on the efficacy of this device must be executed.

### Clinical implications

We demonstrated the feasibility of Probefix in the field of SE. In addition to achieving high quality, stable, hands-free continuous echocardiographic monitoring during exercise, the Probefix has far reaching potential. Continuous monitoring of cardiac function with Probefix could be implemented for the assessment of contractile reserve for response prediction to CRT or for monitoring of myocardial function and hemodynamics under certain physiological or pharmacological conditions [3, 9]. Another application for Probefix could be continuous monitoring of cardiac output at the intensive care department or during cardiac and non-cardiac surgery. A recent case report by our hospital demonstrated for the first time the successful treatment of severe mitral regurgitation through transthoracic echocardiography-guided MitraClip placement [10]. Transcatheter MitraClip placement is traditionally performed using transesophageal echocardiography. However, a history of esophagectomy left the patient involved unfit for transesophageal echocardiography. In special cases like this, the Probefix could facilitate continuous transthoracic echocardiographic measurements. At the same time, the exposure to ionizing radiation of the cardiologist is reduced. As a safe distance to the radiation beam can be secured during measurements.

## Conclusion

In this pilot study, the feasibly of Probefix is demonstrated for stress echocardiography during ergometer tests in supine and upright positions. Although SE with Probefix will not be achievable in all patients due to extensive SE imaging protocols or poor image quality, we believe that the Probefix enables good quality continuous echocardiographic monitoring. Therefore Probefix is able to improve the implementation of an existing diagnostic tool. Validation and scientific proof of the efficacy of this device must be further studied in a larger patient cohort.

## Additional files

Additional file 1: Movie of upright bicycle test with echocardiography using the Probefix. (MP4 6132 kb)

Additional file 2: Movie of supine bicycle test with echocardiography using the Probefix. (MP4 6560 kb)

## Abbreviations

B-mode: Brightness mode
CRT: Cardiac resynchronization therapy
SE: Stress echocardiography
